# Piezo1 and Piezo2 Ion Channels in Neuronal and Astrocytic Responses to MEA Implants in the Rat Somatosensory Cortex

**DOI:** 10.3390/ijms26189001

**Published:** 2025-09-16

**Authors:** Pegah Haghighi, Thomas J. Smith, Ghazaal Tahmasebi, Sophia Vargas, Madison S. Jiang, Ajaree C. Massaquoi, Johnathan Huff, Jeffrey R. Capadona, Joseph J. Pancrazio

**Affiliations:** 1 Department of Bioengineering, The University of Texas at Dallas, Richardson, TX 75080, USA; ghazaal.tahmasebi@utdallas.edu (G.T.); sophia.vargas@utdallas.edu (S.V.); joseph.pancrazio@utdallas.edu (J.J.P.); 2 School of Behavioral and Brain Sciences, The University of Texas at Dallas, Richardson, TX 75080, USA; thomas.smith2@utdallas.edu (T.J.S.); madison.jiang@utdallas.edu (M.S.J.);; 3 Department of Biomedical Engineering, Case Western Reserve University, Cleveland, OH 44106, USA; jrh269@case.edu (J.H.); jrc35@case.edu (J.R.C.); 4 Advanced Platform Technology Center, L. Stokes Cleveland VA Medical Center, Rehabilitation Research and Development, Cleveland, OH 44106, USA

**Keywords:** Piezo1, Piezo2, intracortical microelectrode arrays, neural interface, neural engineering, brain–machine interface

## Abstract

Intracortical microelectrode arrays (MEAs) are tools for recording and stimulating neural activity, with potential applications in prosthetic control and treatment of neurological disorders. However, when chronically implanted, the long-term functionality of MEAs is hindered by the foreign body response (FBR), characterized by gliosis, neuronal loss, and the formation of a glial scar encapsulating layer. This response begins immediately after implantation and is exacerbated by factors such as brain micromotion and the mechanical mismatch between stiff electrodes and soft brain tissue, leading to signal degradation. Despite progress in mitigating these issues, the underlying mechanisms of the brain’s response to MEA implantation remain unclear, particularly regarding how cells sense and respond to the associated mechanical forces. Mechanosensitive ion channels, such as the Piezo family, are key mediators of cellular responses to mechanical stimuli. In this study, silicon-based NeuroNexus MEAs consisting of four shanks were implanted in the rat somatosensory cortex for sixteen weeks. Weekly neural recordings were conducted to assess signal quality over time, revealing a decline in active electrode yield and signal amplitude. Immunohistochemical analysis showed an increase in GFAP intensity and decreased neuronal density near the implant site. Furthermore, Piezo1—but not Piezo2—was strongly expressed in GFAP-positive astrocytes within 25 µm of the implant. Piezo2 expression appeared relatively uniform within each brain slice, both in and around the MEA implantation site across cortical layers. Our study builds on previous work by demonstrating a potential role of Piezo1 in the chronic FBR induced by MEA implantation over a 16-week period. Our findings highlight Piezo1 as the primary mechanosensitive channel driving chronic FBR, suggesting it may be a target for improving MEA design and long-term functionality.

## 1. Introduction

Intracortical microelectrode arrays (MEAs) are tools for recording neural signals at the single-neuron level and stimulating neural activity [1,2], enabling critical insights into brain function and its modulation in both healthy and diseased states [3,4,5]. These devices hold clinical potential for applications, such as prosthetic control [6] and the treatment of neurological disorders [7,8]. However, their long-term reliability is compromised due to, at least in part, the formation of the foreign body response (FBR), a complex process involving gliosis, neuronal loss, and the formation of a dense encapsulating layer around the implant [9,10,11,12,13].

The FBR begins immediately upon MEA insertion [14] and is exacerbated over time by factors such as brain micromotion during chronic implantation [15,16] and the mechanical mismatch between stiff MEAs and the soft brain tissue [17,18]. These factors create a physical and biochemical barrier, reducing electrode functionality and degrading signal quality over time. While progress has been made in mitigating these issues, our understanding of the cellular and molecular paths underlying the brain’s response to MEA implantation remains incomplete. In particular, the mechanisms by which cells sense and respond to the mechanical mismatch associated with MEA chronic implantation require further exploration.

Mechanical stimuli play a vital role in regulating various cellular processes, including migration, proliferation, and survival [19,20,21]. These processes are mediated by mechanosensitive ion channels, which translate mechanical forces into intracellular signaling cascades [22]. Among these channels, the Piezo family has emerged as critical mediators of cellular responses to mechanical stimuli [23,24]. Piezo1 and Piezo2, the two members of this family, are broadly expressed across diverse tissues [25]. Initially, Piezo1 was believed to be predominantly expressed in non-neuronal tissues, while Piezo2 was associated with sensory neurons [23]. This understanding has since evolved, with studies revealing their presence in a wider range of tissues, including the central nervous system (CNS), suggesting they may play critical roles in CNS physiology and pathophysiology [23,26,27,28,29].

Recent studies suggest that Piezo1 may have a role in the FBR [30]. For example, Blumenthal et al. demonstrated that glial scar formation could be mitigated using electrodes with surface nanoroughness [31], an effect mediated by Piezo1 signaling. Similarly, Trotier et al. linked Piezo1 activity to gliosis and inflammation around deep brain electrode implants in the subthalamic nuclei during an eight-week implantation period [30]. Together, these findings suggest that mechanotransduction via Piezo1 may influence both tissue organization and inflammatory activity near MEA implantation sites.

Piezo2, in contrast, has been primarily studied in the peripheral nervous system, where it plays a key role in mechanosensation. While its function in the brain remains poorly understood, recent studies suggest that Piezo2 may also respond to mechanical stimuli in the CNS. For example, Heyburn et al. demonstrated that Piezo2 expression was upregulated following multiple blast exposures, suggesting a potential link between mechanical stress and neurodegeneration [32]. Similarly, Wang et al. showed that Piezo2 channels in the brain can be activated by low-threshold mechanical stimuli [28]. These findings highlight the need for further investigation into the potential role of Piezo2 in mechanotransduction within the brain, particularly in the context involving chronic mechanical stress associated with MEA implantation.

In this study, we demonstrate that during a 16-week chronic implantation period of MEAs in the rat primary somatosensory cortex, Piezo1 was highly expressed in GFAP-positive astrocytes near the implant site. This sustained expression suggests that Piezo1-mediated mechanotransduction may contribute to chronic gliosis and neuroinflammation well into the long-term phase of the FBR. In contrast, Piezo2 showed minimal colocalization with astrocytes, indicating a more neuron-specific and limited role in chronic mechanotransduction.

Overall, these findings broaden the understanding of Piezo channel functions in CNS physiology and inform potential strategies to enhance MEA design and functionality for long-term applications.

## 2. Results

### 2.1. Active Electrode Yield Decays over Time

**Figure 1:**
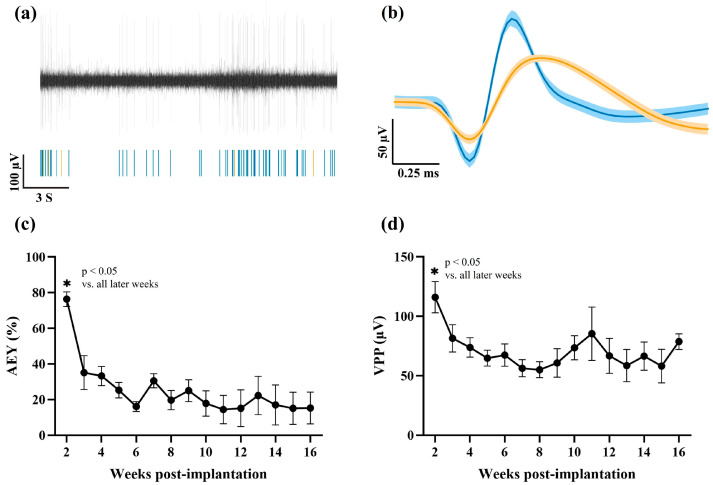
Longitudinal assessment of single-unit activity from chronically implanted MEAs. (**a**) Example raw trace of extracellular voltage recorded from a single channel, with a corresponding raster plot of two detected single units (blue and orange) shown below. (**b**) Average waveforms of the same two isolated single units from the same recording channel, with shaded areas representing the standard error of the mean (SEM). (**c**) Active electrode yield (AEY) over time, expressed as the percentage of electrodes detecting at least one distinguishable unit. (**d**) Peak-to-peak voltage (Vpp) of single-unit waveforms over 16 weeks post-implantation. Data are shown as mean ± SEM. A total of 7 MEAs were used for these recordings. The asterisks indicate significant differences (*p* < 0.05).

Figure 1a,b illustrate the process of detecting single units, while Figure 1c shows the active electrode yield (AEY) calculated based on the detected units. A one-way ANOVA revealed a significant decline in AEY following week 2, with all subsequent weeks showing significantly lower values (*p* < 0.05). Two weeks post-implantation, the AEY of the implanted MEAs (n = 7) was 76 ± 4%, but it dropped below 50% by week 3 post-implantation, eventually declining to 15 ± 9% by week 16.

We also examined the Vpp of single units recorded. We observed a decline in the average peak-to-peak voltage (*p* < 0.05), beginning at 116 ± 13.0 μV two weeks post-implantation, and an amplitude of 79 ± 7.0 μV by the end of the study.

These results are consistent with previous findings that chronic implantation of silicon MEAs leads to a progressive decline in recording quality due to the foreign body response and tissue encapsulation [9,11]. Notably, prior work from our own lab has demonstrated that other device types—such as single-shank silicon probes and microwire arrays—tend to maintain higher AEY over time [33,34]. Compared to those platforms, the rapid signal degradation observed here may reflect device-specific differences in geometry, material stiffness, tissue response to multiple shanks, or differences in force required for insertion.

### 2.2. Reactive Astrocytes Are Elevated near the Implant Site 16 Weeks Post-Implantation

**Figure 2:**
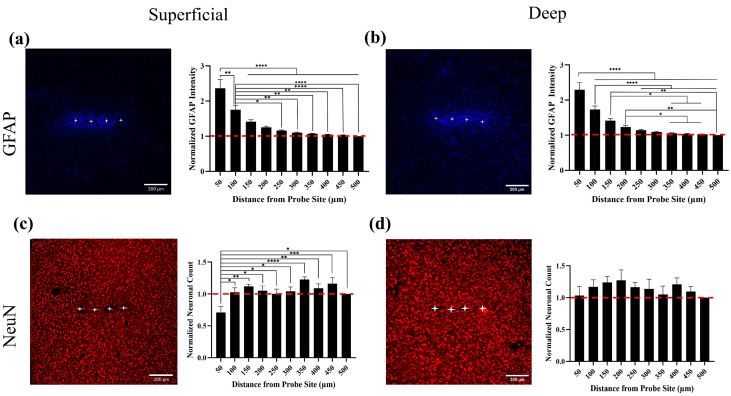
Spatial analysis of astrocytic activation and neuronal density in superficial and deep cortical layers surrounding the implant site. (**a**,**b**) Representative GFAP immunofluorescence images (blue) in superficial (**a**) and deep (**b**) cortical layers, alongside quantification of normalized GFAP intensity as a function of distance from the implant site. The GFAP signal was highest at 50 µm and significantly greater than all other distances (*p* < 0.05), with 100 µm also elevated compared to 250 µm and beyond. (**c**,**d**) Representative NeuN-labeled images (red) from the superficial (**c**) and deep (**d**) cortex, with corresponding quantification of normalized NeuN-positive cell counts. In the superficial cortex, neuronal density at 50 µm was significantly lower than at all other distances (*p* < 0.05). No significant differences were detected in deep layers. All data are shown as mean ± SEM across 8 animals. Fluorescence intensity and cell counts were normalized to values from the farthest ROI (500 µm). Statistical analysis was performed using one-way ANOVA followed by Tukey’s post hoc test. * *p* < 0.05, ** *p* < 0.01, *** *p* < 0.001, and **** *p* < 0.0001. Red dashed lines represent the normalized baseline (1.0). White asterisks indicate the locations of implant holes. Scale bars: 200 µm.

Consistent with prior work [35,36,37,38,39], quantification of GFAP fluorescence intensity revealed a distance-dependent decrease in astrocytic activation surrounding the implant (Figure 2a,b). In superficial cortical layers (0–800 µm), GFAP intensity at 50 µm from the implant site was significantly higher than all other measured distances (*p* < 0.05). Intensity at 100 µm was also significantly elevated compared to 250 µm and farther. A similar pattern was observed in deep cortical layers (800–1600 µm), where the 50 µm region showed significantly higher GFAP intensity than all other distances, and 100 µm was significantly greater than 250 µm and beyond. These results indicate a pronounced and spatially confined astrocytic response near the implant site, diminishing with distance.

### 2.3. Localized Neuronal Loss in Superficial Cortex

Normalized NeuN-positive cell counts revealed a reduction in neuronal density near the implant site, primarily in superficial layers (Figure 2c,d). At 50 µm, neuronal density was significantly lower than at all other distances except 250 µm (*p* < 0.05). In deep cortical layers, there were no significant differences in NeuN counts across distances, suggesting that neuronal loss or displacement is largely confined to the superficial region adjacent to the implant. This observation is consistent with prior studies [34,37,38], which have shown that the foreign body response and associated neuronal disruption are most pronounced near the cortical surface and diminish with depth.

### 2.4. Piezo1 Shows Elevated Expression near the Implant Site Compared to Piezo2

**Figure 3:**
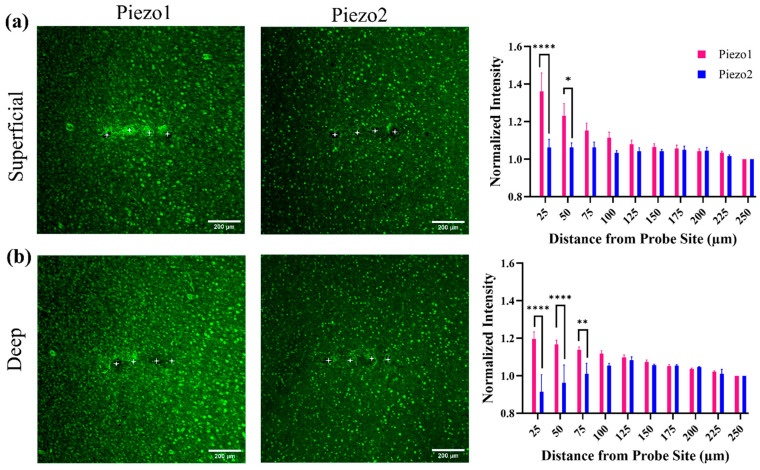
Differential spatial distribution of Piezo1 and Piezo2 expression around the implant site in the superficial and deep cortex. (**a**) Representative 10× immunofluorescence images of Piezo1 (left) and Piezo2 (middle) expression in the superficial cortex, with corresponding quantification of normalized intensity (right). (**b**) Similar images and quantification from the deep cortex. White asterisks indicate the implant holes. Piezo1 intensity was significantly higher than Piezo2 within 25–75 µm from the implant in both cortical layers. Piezo2 expression remained stable across all distances. Data are shown as mean ± SEM. Seven animals were used for Piezo1 analysis, while Piezo2 analysis included three animals for the superficial layers and two animals for the deep layers. Fluorescence intensity values were normalized to the most distal ROI (250 µm). Statistical analysis was performed using two-way ANOVA followed by Tukey’s post hoc test. * *p* < 0.05, ** *p* < 0.01, and **** *p* < 0.0001 Scale bars: 200 µm.

Fluorescence intensity analysis revealed distinct spatial expression patterns for Piezo1 and Piezo2 surrounding the implant site (Figure 3). In both superficial and deep cortical layers, Piezo1 expression was highest within 25 µm of the implant and significantly greater than Piezo2 at the same distance (*p* < 0.0001). Piezo1 intensity gradually decreased with distance, with significant differences between 25 and 75 µm (*p* < 0.01), and between 25 and 100 µm (*p* < 0.05), indicating a localized peri-implant enrichment. In contrast, Piezo2 expression remained relatively uniform across distances and did not show a strong spatial gradient. The same pattern was observed in both superficial and deep layers, suggesting that Piezo1—but not Piezo2—may be selectively upregulated near the chronic implant.

### 2.5. Piezo1 Preferentially Colocalizes with Astrocytes near the Implant Site

**Figure 4:**
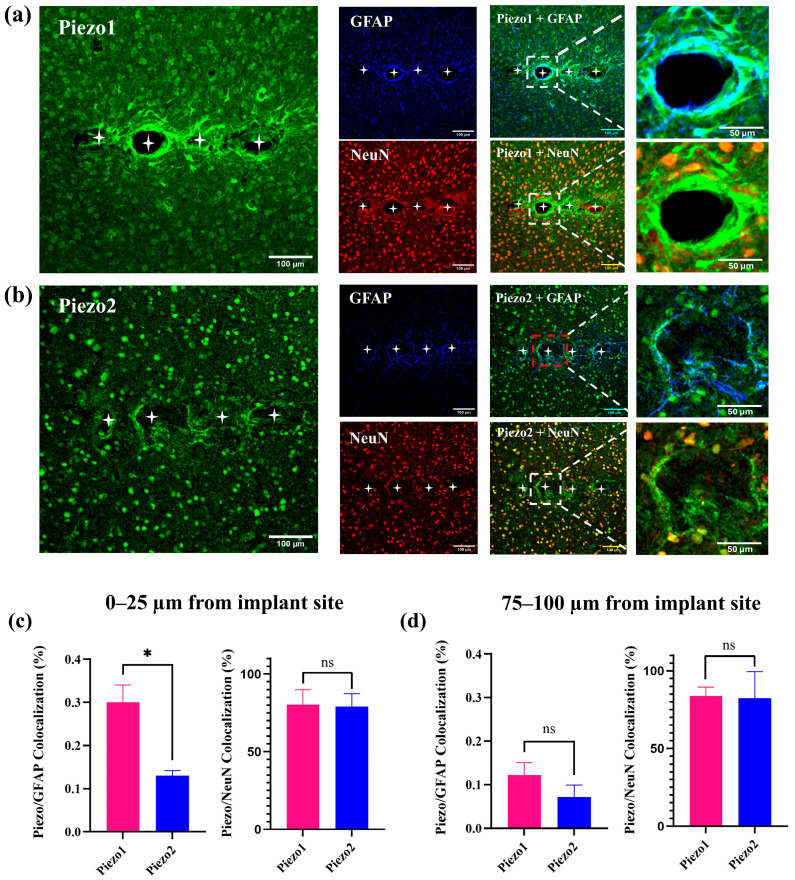
Colocalization of Piezo1 and Piezo2 with GFAP and NeuN at varying distances from the implant site. Representative immunofluorescence images of cortical tissue surrounding chronically implanted MEAs showing expression of (**a**) Piezo1 and (**b**) Piezo2 channels (green), co-labeled with GFAP (astrocytes, blue) and NeuN (neurons, red). Merged panels illustrate colocalization of Piezo channels with GFAP (cyan) and with NeuN (orange), with high-magnification insets highlighting peri-implant regions (scale bars: 100 µm for low magnification; 50 µm for insets). Quantification of colocalization at (**c**) 0–25 µm and (**d**) 75–100 µm from the implant site shows significantly greater Piezo1–GFAP colocalization compared to Piezo2–GFAP within 25 µm of the implant (* *p* < 0.05), while Piezo–NeuN colocalization did not differ between Piezo1 and Piezo2. Data are presented as mean ± SEM; ns = not significant. White asterisks represent the location of implant holes.

To assess the spatial association of mechanosensitive channels with glial and neuronal markers, we performed colocalization analysis of Piezo1 and Piezo2 with GFAP and NeuN at two distances from the implant site: 0–25 µm (proximal) and 75–100 µm (distal; Figure 4). In the proximal region, Piezo1 showed a significantly greater colocalization percentage with GFAP compared to Piezo2 (*p* < 0.05), suggesting enhanced astrocytic interaction near the probe–tissue interface. No significant difference was observed in Piezo1 vs. Piezo2 colocalization with NeuN-positive cells at this distance.

In the distal region (75–100 µm), no significant differences were detected between Piezo1 and Piezo2 in colocalization with either GFAP or NeuN. These findings suggest that Piezo1 is preferentially associated with reactive astrocytes in the immediate vicinity of the implant, while both channels exhibit similar distribution patterns at farther distances.

## 3. Discussion

This study provides new insights into the spatial expression and cellular localization of Piezo1 and Piezo2 channels in the context of chronic MEA implantation in the rat somatosensory cortex. Through spatial analysis at 16 weeks post-implantation, declining electrode performance is associated with expression patterns of these mechanosensitive ion channels. Specifically, we observed marked reductions in AEY and spike amplitude, where spike amplitude (Vpp) reflects the extracellular action potential magnitude and serves as a proxy for neuron–electrode proximity and recording quality. Larger Vpp values generally indicate that neurons are closer to or better coupled with the electrode, whereas declining amplitudes suggest increased distance, neuronal loss, or glial encapsulation [40]. This is consistent with previous reports attributing such decline to FBR-associated gliosis, inflammation, and neuronal loss. The observed decline in active electrode yield (AEY) over time was more rapid than previously reported AEY profiles from four-shank silicon-based probes used in our lab [37,38]. Notably, by week 2 post-implantation, AEY had already dropped below 50%, reaching only 15 ± 9% SEM by week 16. This accelerated decline may be attributed, in part, to the physical characteristics or insertion force dynamics of the multi-shank MEAs used in this study. The A4x4-3mm-100-125-703-CM16LP probes used here have longer shanks (3 mm), closer shank spacing (125 µm), and larger recording site areas (703 µm^2^) compared to other NeuroNexus probes, such as the A4x4-2mm-200-200-200-CM16LP used in the previous studies. These larger and denser probe dimensions could result in greater insertion-related damage and a more robust foreign body response, thereby contributing to the faster loss of recording capability observed in our data.

Although Piezo1 has been well-characterized in peripheral tissues and endothelial cells [41,42,43,44], few studies have explored its spatial distribution in the CNS, especially in the context of implanted neural interfaces. Our data demonstrate that Piezo1 expression is highest within 25 µm of the implant site and colocalizes with GFAP-positive astrocytes. This observation aligns with prior studies suggesting that Piezo1 mediates glial reactivity to mechanical cues. Notably, Piezo1 activation in astrocytes has been shown to promote cytokine release and neuroinflammation [45], processes that disrupt neuronal–astrocytic homeostasis and impair signal fidelity. Beyond astrocytic reactivity, Piezo1 has also been shown to disrupt blood–brain barrier integrity via CaMKII/Nrf2 signaling during ischemic stroke [46]. These findings suggest that Piezo1 activation in the CNS can initiate broader pathological cascades, potentially overlapping with the chronic neuroinflammatory responses we observed at MEA implant sites. Our findings extend these observations by demonstrating a spatially confined enrichment of Piezo1 expression in glial cells surrounding MEAs at chronic time points. It should be noted, however, that Piezo1 staining was not exclusive to astrocytes—other cell populations (e.g., microglia) may also contribute to the peri-implant Piezo1 signal, though these were not specifically probed in our study. Our results are further supported by parallel findings in the motor cortex, where Piezo1 expression remains elevated at 2, 8, and 16 weeks post-implantation (Supplementary Methods and Results S1) [47,48], reinforcing its sustained involvement in the FBR. Emerging evidence also suggests that Piezo1’s function is not solely mechanically driven but may also be modulated by biochemical factors. For instance, fatty acids have been identified as modulators of Piezo1 in astrocytes, influencing cytokine release and gliosis in models of Alzheimer’s disease [49]. This dual sensitivity to mechanical and biochemical stimuli highlights the complexity of Piezo1 signaling in neuroinflammatory pathways and positions it as a potential molecular target to perhaps mitigate chronic glial activation near neural implants.

In contrast, Piezo2 displayed relatively uniform expression across cortical depths and distances from the implant, with no significant spatial gradient or astrocytic colocalization. This is consistent with prior reports suggesting that Piezo2 functions predominantly in neuronal mechanosensation and network synchronization [28], rather than in reactive glial responses. Although Piezo2 expression has been documented in astrocytes in certain contexts, such as the optic nerve head [29], our findings suggest a limited role for Piezo2 in the chronic FBR surrounding intracortical MEAs.

Taken together, our results suggest a spatially restricted, glia-associated role for Piezo1 during chronic neural interface implantation, in contrast to the more uniform and neuron-associated expression of Piezo2. These findings raise the possibility that Piezo1-mediated mechanotransduction is associated with glial remodeling and neuroinflammatory signaling near the implant surface, though it remains unclear whether Piezo1 plays a causal role or serves as a marker of the chronic foreign body response. By extending previous observations to a 16-week chronic time point and incorporating spatially resolved colocalization analysis, this study adds important temporal and anatomical context to Piezo1’s involvement in the FBR. It should be noted that our study did not include intermediate histological time points between implantation and 16 weeks. Future investigations incorporating earlier time points could help clarify how the rapid early decline in AEY may influence subsequent Piezo1 expression patterns and peri-implant glial responses. Future studies may also investigate whether targeted modulation of Piezo1—either through mechanical design of MEAs or pharmacological intervention—can attenuate glial activation and prolong electrode functionality. Additionally, further exploration of Piezo2 under conditions of acute injury, elevated intracranial pressure, or neurodegeneration may clarify its roles in CNS mechanotransduction.

Beyond biological characterization, our findings also intersect with engineering strategies for mitigating chronic tissue responses. The probes used in this study are relatively stiff (flexural rigidity~2.8 × 10^−8^ N·m^2^), and prior work has shown that higher stiffness strongly correlates with glial scarring and neuronal loss near the implant [50]. Finite-element and experimental studies have demonstrated that the large mechanical mismatch between silicon probes and brain tissue generates significant interfacial strain, which can contribute to chronic tissue damage [51]. Bare silicon shanks have been reported to impose dynamic strains of 2.5–3.5% on surrounding brain tissue, whereas softer, compliant materials, such as PVAc-coated silicon or nanocomposites, reduce interfacial strain to ≤1.5%. Our multi-shank silicon probes (100 × 50 µm^2^, cross-sectional area 5000 µm^2^) are larger and stiffer than the single-shank silicon probes studied in these prior works (123 × 15 µm^2^, cross-sectional area 1845 µm^2^) and are, therefore, likely to generate even higher interfacial strains, potentially contributing to the accelerated decline in electrode performance and increased chronic foreign body response observed in our study. Given our observation of Piezo1 upregulation in peri-implant astrocytes, it is plausible that mechanical strain from stiff devices engages Piezo1-mediated mechanotransduction pathways, driving glial remodeling and neuroinflammation. Architectural solutions, such as thinner, more flexible shanks or alternative materials (e.g., a-SiC or polymer-based MEAs), may, therefore, attenuate Piezo1 activation and improve chronic performance. These observations are consistent with recent perspectives highlighting Piezo channels as central mediators of mechano-inflammation and neuroimmune crosstalk [52], suggesting that strategies targeting Piezo1 activity may mitigate chronic glial activation at neural interfaces. Overall, our findings contribute to a growing understanding of mechanosensitive signaling in the brain and offer new directions for optimizing neural interface design and mitigating long-term tissue responses.

## 4. Materials and Methods

### 4.1. Ethics Statement

All animal care, housing, and experimental procedures were conducted in accordance with the guidelines approved by the Institutional Animal Care and Use Committee (IACUC) at the University of Texas at Dallas (protocol #21-15).

### 4.2. Animal Use and MEA Implantation

A total of eight male Sprague–Dawley rats (n = 8; Charles River Laboratories, Houston, TX, USA) were used in this study. Animals were single housed in standard cages under a reverse 12 h light/dark cycle (lights off at 6 a.m.) with ad libitum access to water. To promote behavioral engagement, food was restricted to maintain ≥90% of free-feeding body weight on training days, with free-feeding permitted on weekends. Reward pellets (F0021, Bio-Serv, Flemington, NJ, USA) were used as conditioned reinforcers during behavioral sessions, and supplemental chow (5LL2-Prolab^®^ RMH 1800, LabDiet, St. Louis, MO, USA) ensured balanced nutrition. Animals falling below the weight criteria or failing to respond to control stimuli were temporarily excluded until recovery.

**Figure 5:**
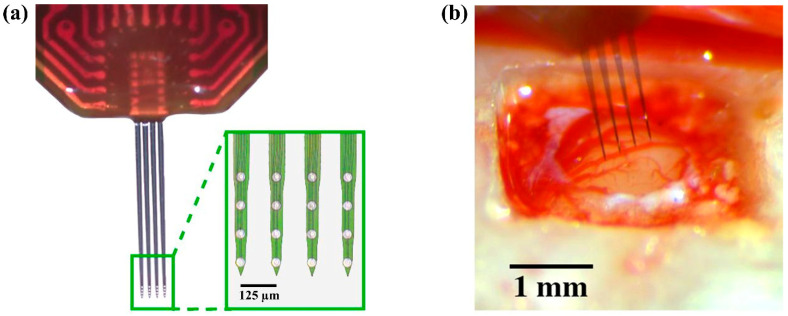
Neural probe used for chronic implantation. (**a**) Image of a four-shank silicon-based intracortical microelectrode array (MEA) used in this study. Each shank contains four recording sites. Inset shows a magnified view of the electrode sites spaced along the shanks (scale bar: 125 µm). (**b**) Image of the MEA positioned above the cortical surface prior to insertion (scale bar: 1 mm).

Surgical implantation was performed under aseptic conditions, as previously described [53]. Briefly, animals were anesthetized with isoflurane (1.8–2.5%), placed in a stereotaxic frame, and given local and systemic analgesia. After scalp incision and craniotomy over S1FL, a four-shank silicon MEA (Figure 5; A4x4–3 mm–100–125–703-CM16LP, NeuroNexus Technologies Inc., Ann Arbor, MI, USA) was lowered to the target 2 mm depth using a precision inserter. The array was secured with screws and dental cement, the wound was closed, and animals received antibiotic prophylaxis, analgesics, and postoperative monitoring prior to resuming behavioral training.

Following MEA implantation and recovery, animals were handled and habituated before undergoing intracortical microstimulation (ICMS) and behavioral training, as previously described [54]. ICMS was delivered via a PlexStim external stimulator using monopolar, charge-balanced, biphasic pulses (200 µs/phase, 40 µs interphase delay) at 320 Hz, presented as 650 ms trains. Current amplitudes ranged from 0 to 100 µA, with naive thresholds established 7–12 days post-implantation and confirmed by paw withdrawal. Animals were trained in a multi-stage, two-choice discrimination paradigm, where correct responses were rewarded with sugar pellets and incorrect responses were followed by mild air-puff punishments. Experimental and retraining phases alternated to maintain task performance as new ICMS patterns were introduced.

Since there was no significant difference in Piezo1 expression between the control and stimulated groups, data from both cohorts were combined for subsequent analyses (Supplementary Figure S1).

### 4.3. Electrophysiological Recordings and Data Processing

Neural recording sessions, each lasting 10 min, were conducted weekly on awake animals placed inside a Faraday cage. Wideband signals from the primary somatosensory cortex were collected from all 16 electrodes of the MEAs simultaneously using the OmniPlex^®^ Neural Recording Data Acquisition System (Plexon, Inc., Dallas, TX, USA) at a sampling rate of 40 kHz.

Using Plexon Offline Sorter software (×64 V4.7.1, Plexon Inc., Dallas, TX, USA), the continuous wideband data were processed. Initially, a four-pole Butterworth bandpass filter (300–3000 Hz) was applied to the wideband recordings. Channels were then normalized to the median signal of all channels to minimize the impact of noise and artifacts [55]. Potential single-unit waveforms were identified as those exceeding −4 standard deviations from the baseline. Automated clustering of waveforms in principal component space was performed using k-means, followed by manual verification to confirm single-unit identification. Active electrode yield (AEY) was calculated as the percentage of functional channels detecting at least one unit per session. Although eight animals were implanted, one was excluded from analysis due to persistent signal loss and a lack of discernible units in early recordings. Immunohistological analysis of Piezo1 and GFAP expression patterns in this animal were within the range of the other rats and did not represent an outlier (Supplementary Figure S2). Recordings were re-attempted in subsequent weeks but consistently failed to yield stable units, likely due to factors like poor connection at the head stage, bond pad failure, or other signal pathway issues, confirming the early exclusion from electrophysiological analysis.

### 4.4. Immunohistochemistry

Immunohistochemical processing of rat brains with implanted MEAs was carried out as follows: Rats were euthanized 16 weeks post-implantation with an intraperitoneal injection of sodium pentobarbital (Virbac Corporation, Westlake, TX, USA) and transcardially perfused with 1× PBS, followed by 4% paraformaldehyde (PFA; Sigma-Aldrich, St. Louis, MO, USA). The skulls were then immersed in 4% PFA for 48 h before the MEAs were removed. Brains were carefully extracted and stored in PBS containing sodium azide for 24 h at 4 °C before histological processing. Then, tissue was mounted on the vibratome stage and sectioned into 100 µm-thick slices. The slices were treated with sodium borohydride (Sigma-Aldrich, MO, USA) for 30 min to reduce autofluorescence, followed by three 20-min washes with 1× PBS. The sections were then blocked in a blocking buffer containing 4% normal goat serum (Abcam Inc., Waltham, MA, USA), 0.3% Triton X-100 (Sigma-Aldrich, MO, USA), 0.1% sodium azide (Sigma-Aldrich, St. Louis, MO, USA), and 2% bovine serum albumin (Sigma-Aldrich, St. Louis, MO, USA) for one hour. After blocking, the sections were washed three times with 1× PBS containing 0.1% sodium azide and incubated with Image-iT (Thermo Fisher Scientific, Waltham, MA, USA) for 30 min. The slices were then washed three times with 1× PBS containing 0.1% sodium azide, followed by overnight incubation at 4 °C with primary antibodies diluted in the blocking buffer.

On the following day, the slices were washed for 15 min with 1× PBS, then incubated for 15 min in 1× PBS containing 0.2% Triton X-100 and washed again with 1× PBS for 15 min. The sections were then incubated with secondary antibodies diluted in the blocking buffer for 2 h. After secondary antibody incubation, the sections were washed again with 1× PBS for 15 min, followed by 15 min in 1× PBS containing 0.2% Triton X-100, and a final 15-min wash in 1× PBS. The slices were then mounted onto glass slides using fluoromount aqueous mounting medium (Sigma-Aldrich, St. Louis, MO, USA) and cover-slipped. The specific antibodies used are listed in Table 1. It should be noted that both Piezo1 and Piezo2 antibodies used in this study are polyclonal. Their specificity has been supported by secondary-only controls (Supplementary Figure S3) and prior publications. The specificity of the Piezo2 antibody (Novus Biologicals, Centennial, CO, USA, NBP1-78624; RRID: AB_11005294) has been demonstrated by Murthy et al. [56], who showed that Piezo2 interacts with E-cadherin in specialized gastrointestinal epithelial mechanoreceptors using immunofluorescence and immunoprecipitation assays. The specificity of the Piezo1 antibody (Novus Biologicals, NBP1-78537; RRID: AB_11005294) has been validated using a genetic knockdown approach (siRNA) in primary cilia [57]. While these studies strongly support antibody specificity, we cannot completely rule out potential cross-reactivity.

### 4.5. Image Analysis

Images were captured using an Olympus FV4000RS laser confocal microscope at 10 and 20× magnification. A maximum intensity projection was generated from a 35 µm z-stack for each slice. All imaging parameters were standardized across samples to ensure consistency.

Fluorescent images of brain slices collected from animals implanted with microelectrode arrays were analyzed to quantify the intensity of GFAP, Piezo1, and Piezo2 expression, neuronal density around the implant site, as well as Piezo1/2 colocalization with GFAP and neurons.

For GFAP intensity analysis, 10× magnification images were used. A custom MATLAB (R2024b, 24.2) script was employed to define rectangular regions of interest (ROIs) measuring 50 × 100 µm, extending from the edge of the implantation site up to 500 µm both above and below the site. Mean fluorescence intensity was extracted from each ROI, and the values were normalized to the intensity measured in the most distal ROI. Slices were categorized into superficial (0–800 µm depth) and deep (800–1600 µm depth) cortical layers. Data from 8 animals were included in the analysis. The normalized intensity values at each distance were averaged across animals and plotted. One-way ANOVA was used to evaluate the statistical significance of intensity differences across distances.

For Piezo1 and Piezo2 intensity analyses, images acquired at 20× magnification were used. The same MATLAB-based approach was applied using smaller ROIs measuring 25 × 100 µm, extending up to 250 µm from the implant cavity. As with GFAP, intensity values were normalized to the farthest ROI and grouped into superficial and deep layers. A total of seven animals were used for Piezo1 analysis and three animals for Piezo2 at the superficial layers and two animals at the deep layers. Two-way ANOVA was used to assess differences in intensity across distances and between Piezo1 and Piezo2 channels.

For neuronal quantification, 10× images were analyzed using 50 × 100 µm ROIs placed serially above and below the implant cavity. Neurons were manually counted within each ROI. To assess distribution as a function of distance, the number of neurons in adjacent ROIs (e.g., 0–50 µm above and below, 50–100 µm, etc.) were summed cumulatively and then normalized to the count in the farthest ROI. This allowed evaluation of relative neuronal density at increasing distances from the implant. Eight animals were used for this analysis. One-way ANOVA was performed to assess differences in normalized neuronal density across distances.

Colocalization analyses were conducted in FIJI (ImageJ 1.54p, Java 21.0.4, National Institutes of Health, Bethesda, MD, USA). For both Piezo1 and Piezo2, slices were selected from within the first 1000 µm along the implant tract. For Piezo1, slices showing the strongest peri-implant expression were prioritized to ensure a sufficient signal for analysis. For Piezo2, which exhibited more uniform expression, a subset of slices was excluded if they contained excessive noise or poor signal quality to avoid skewing the colocalization results. Binary masks were generated for Piezo1, Piezo2, GFAP, and NeuN using Costes’ automatic thresholding. Colocalization images were created using the Image Calculator to generate Piezo1_GFAP, Piezo2_GFAP, Piezo1_NeuN, and Piezo2_NeuN masks.

Within each selected image, two 25 × 100 µm ROIs were placed on one side of the implant site: one at the region closest to the implant (0–25 µm) and one farther away (75–100 µm). The proximal ROI was chosen based on the area with the highest Piezo expression that also contained NeuN-positive cells, ensuring that colocalization analysis was conducted in biologically relevant regions. For astrocytic colocalization, the pixel area of the Piezo_GFAP mask was divided by the total Piezo pixel area in each ROI to calculate the percentage of overlap. For neuronal colocalization, NeuN-positive cells and Piezo_NeuN-positive cells were manually counted in each ROI, and the percentage of colocalization was calculated as the number of colocalized cells divided by the total number of NeuN-positive cells.

All statistical analyses were conducted using GraphPad Prism (10.4.1 (627), Dotmatics, Boston, MA, USA). One-way ANOVA was used for GFAP intensity and neuronal counts, and two-way ANOVA was applied for comparison of Piezo1 and Piezo2 intensities. When applicable, Tukey’s post hoc tests were performed. Significance was defined as *p* < 0.05. Data are reported as mean ± SEM.

## Figures and Tables

**Table 1:** Antibody list used for IHC staining.

Antibody	Target	Concentration	Supplier	Catalog Number
NeuN	Neuronal Nuclei	1:500	Abcam	ab4674
GFAP	Astrocytes	1:500	Abcam	ab104224
Piezo1	Piezo1 channels	1:50	Novus Biologicals	NBP2-10504
				RRID: AB_3252279
Piezo2	Piezo2 channels	1:100	Novus Biologicals	NBP178624SS
				RRID: AB_11005294
Alexa Fluor 555	NeuN	1:4000	Abcam	ab150118
Alexa Fluor 647	GFAP	1:4000	Abcam	ab150171
Alexa Fluor 488	Piezo1/2	1:2000	Invitrogen (Carlsbad, CA, USA)	A11034

## Data Availability

The data presented in this study are available upon request from the corresponding author. The data are not publicly available due to ethical restrictions.

## Supplementary Materials

The following supporting information can be downloaded at: https://www.mdpi.com/article/10.3390/ijms26189001/s1.
