# Supplementary material for: Piezo1 and Piezo2 Ion Channels in Neuronal and Astrocytic Responses to MEA Implants in the Rat Somatosensory Cortex
Source: Int J Mol Sci. 2025 Sep 16;26(18):9001. doi: 10.3390/ijms26189001 (PMC12469562; doi:10.3390/ijms26189001)
Supplement: Supplementary file 1 [file ijms-26-09001-s001.zip › ijms-3848069-supplementary.pdf]

# Supplemental:

## Supplementary Methods and Results S1:

**Gene expression method:**

Sprague-Dawley rats were implanted with custom-made silicon single-shank probes from Qualia Labs (2 mm long, 1.5 mm shank, 123 µm wide, 15 µm thick) in the motor cortex following our previously published protocol [47]. The probes were sterilized using ethylene oxide gas at 54.4 °F with a 1-hour sterilization cycle and 12-hour aeration before being stored in a sealed container until surgery.

The study included three time points: 2 weeks post-implantation (n = 4), 8 weeks (n = 3), and 16 weeks (n = 4).

RNA extraction and bulk analysis followed a previously published protocol [48] with key modifications. Specifically, a custom code set of 218 genes, including six housekeeping genes and 212 target genes, was used. Additionally, Nanostring Technologies, now Bruker Spatial Biology (Tucson, AZ, United States), was utilized for the analysis.

For statistical analysis, gene count data were processed using nSolver. Normalization was performed using positive controls and housekeeping genes. Log2 fold change comparisons were conducted for each condition and time point relative to naïve control tissue (animals with no surgical procedure). Statistical significance was determined using unpaired t-tests with Benjamini-Hochberg correction, and genes with adjusted $p < 0.05$ were considered significant.

**Results:**

To investigate the temporal regulation of PIEZO1 and PIEZO2 following implantation, we performed bulk RNA analysis at 2, 8, and 16 weeks post-implantation. Our results show that PIEZO1 was significantly upregulated at all three time points, with a 1.3-fold increase at 2 weeks ($p$ = 4.47E-05), a moderate increase at 8 weeks (Log2 = 0.683, $p$ = 0.00405), and a rise again at 16 weeks (Log2 = 1.03, $p$ = 4.89E-06).

In contrast, Piezo2 was not expressed at any of the examined time points, suggesting that Piezo1**,** but not Piezo2, may play a role in the tissue response following probe implantation**.** The transient decrease in Piezo1 expression at 8 weeks could indicate a dynamic regulation of mechanosensitive ion channels in response to chronic implantation.

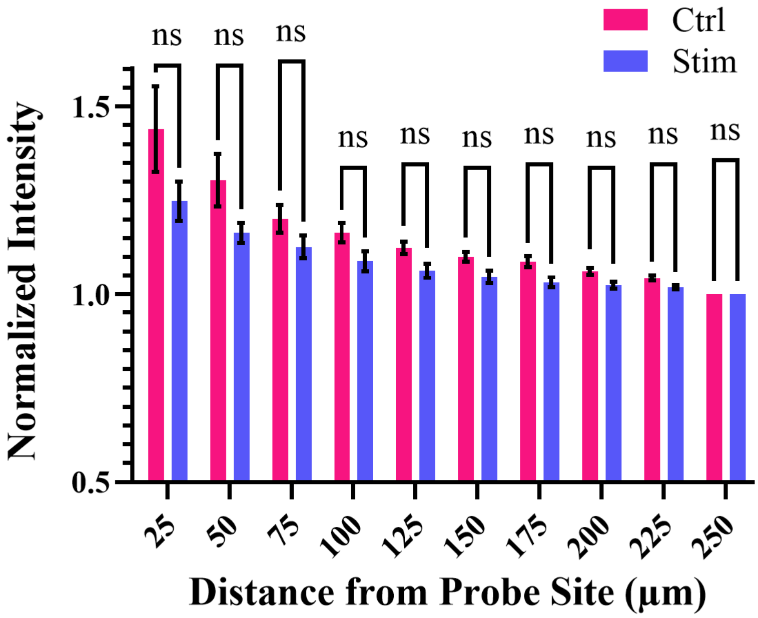


Figure S1: Comparison of normalized Piezo1 expression intensity in control (Ctrl, magenta, n=4) and stimulated (Stim, blue, n=3) groups at increasing distances from the probe site (25–250 µm). Data are shown as mean ± SEM. Two-way ANOVA with significance threshold set at $P < 0.05$ revealed no significant differences (ns) between groups at any distance, supporting the combination of both cohorts for subsequent analyses.

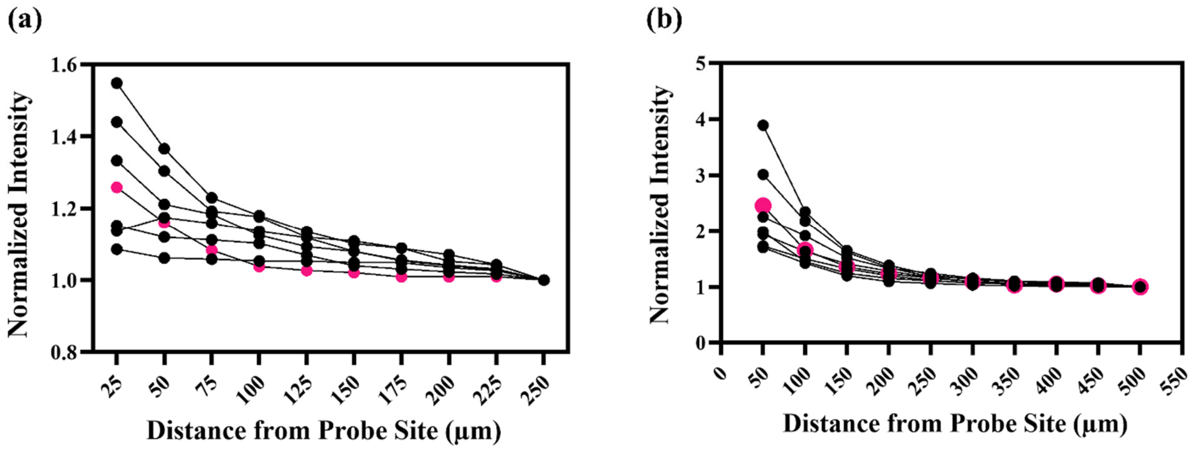


Figure S2. Individual animal plots of normalized intensity as a function of distance from the probe site. (a) Piezo1 and (b) GFAP expression. Each black trace represents a single animal, and the pink trace corresponds to the animal excluded from electrophysiological analysis due to persistent signal loss. ROUT test analysis confirmed that this animal's histological data were not statistical outliers and fell within the range of the other animals.

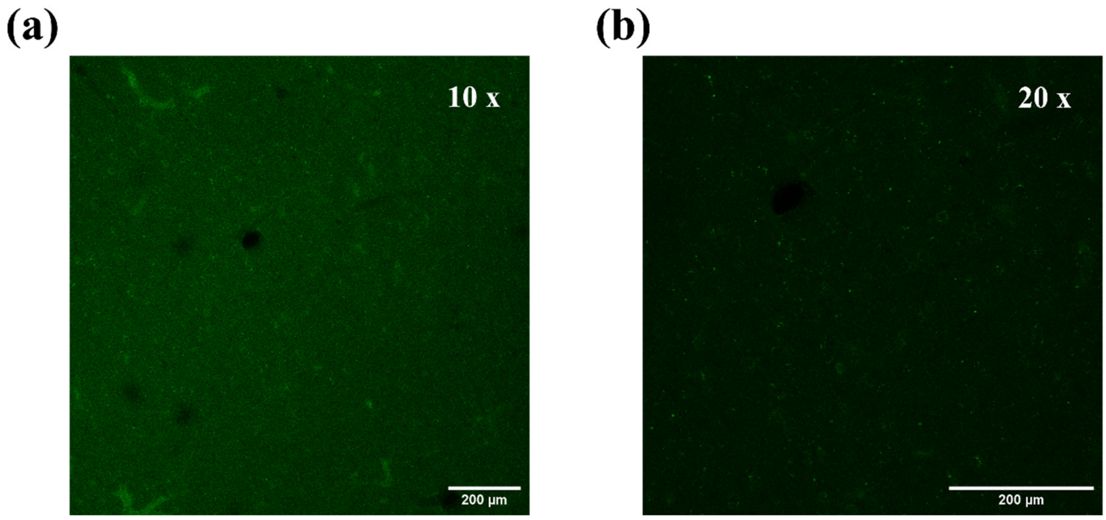


Figure S3. Validation of Piezo1 and Piezo2 immunostaining. (a) Secondary antibody-only control for Alexa Fluor 488, corresponding to Piezo1 or Piezo2 primary antibodies, imaged at 10× magnification. Scale bar: 200 µm. (b) Higher-magnification view of the same region at 20× magnification. Images show minimal background fluorescence, confirming specificity of the primary antibodies.
